# Pharmaceutical expenditure on drugs for rare diseases in Canada: a historical (2007–13) and prospective (2014–18) MIDAS sales data analysis

**DOI:** 10.1186/s13023-016-0450-y

**Published:** 2016-05-21

**Authors:** Victoria Divino, Mitch DeKoven, Michael Kleinrock, Rolin L. Wade, Tony Kim, Satyin Kaura

**Affiliations:** IMS Health, 8280 Willow Oaks Corporate Drive, Suite 775, Fairfax, VA 22031 USA; IMS Institute for Healthcare Informatics, One IMS Drive, Plymouth Meeting, PA 19462 USA; Celgene, 6755 Mississauga Rd, Suite 600, Mississauga, ON L5N 7Y2 Canada; Celgene, 86 Morris Avenue, H-222H, Summit, NJ 07901 USA

**Keywords:** Orphan drugs, Health economics, Cost of health care, Rare diseases, Pharmaceuticals, Health spending

## Abstract

**Background:**

Health Canada has defined rare diseases as life-threatening, seriously debilitating, or serious chronic conditions affecting a very small number of patients (~1 in 2,000 persons). An estimated 9 % of Canadians suffer from a rare disease. Drugs treating rare diseases (DRDs) are also known as orphan drugs. While Canada is currently developing an orphan drug framework, in the United States (US), the Orphan Drug Act (ODA) of 1983 established incentives for the development of orphan drugs.

This study measured total annual expenditure of orphan drugs in Canada (2007–13) and estimated future (2014–18) orphan drug expenditure.

**Methods:**

Orphan drugs approved by the US Food and Drug Administration (FDA) in the US were used as a proxy for the orphan drug landscape in Canada. Branded, orphan drugs approved by the FDA between 1983 through 2013 were identified (*N* = 356 unique products). Only US orphan drugs with the same orphan indication(s) approved in Canada were included in the analysis. Adjustment via an indication factoring was applied to products with both orphan and non-orphan indications using available data sources to isolate orphan-indication sales. The IMS Health MIDAS database of audited biopharmaceutical sales was utilized to measure total orphan drug expenditure, calculated annually from 2007–2013 and evaluated as a proportion of total annual pharmaceutical drug expenditure (adjusted to 2014 CAD).

**Results:**

Between 2007 and 2013, expenditure was measured for a final *N* = 147 orphan drugs. Orphan drug expenditure totaled $610.2 million (M) in 2007 and $1,100.0 M in 2013, representing 3.3– 5.6 % of total Canadian pharmaceutical drug expenditure in 2007–2013, respectively. Future trend analysis suggests orphan drug expenditure will remain under 6 % of total expenditure in 2014–18.

**Conclusions:**

While the number of available orphan drugs and associated expenditure increased over time, access remains an issue, and from the perspectives of society and equity, overall spending on orphan drugs is lower relative to the number of patients affected with an orphan disease in Canada. The overall budget impact of orphan drugs is small and fairly stable relative to total pharmaceutical expenditure. Concerns that growth in orphan drug expenditure may lead to unsustainable drug expenditure do not appear to be justified.

## Background

Health Canada has defined a rare disease as a life-threatening, seriously debilitating, or serious and chronic condition affecting a relatively small number of patients: 1 in 2,000 persons [1]. It is estimated that 1 in 12 Canadians, approximately 3 million people or 9 % of the Canadian population, suffer from a rare disease [2]. Drugs that have been developed to treat rare diseases, also known as drugs treating rare diseases (DRDs), are designated as orphan drugs if certain criteria are met, such as criteria regarding prevalence or severity of the condition, as well as the lack of existing treatments or significant improvement compared to the standard drug treatment [1].

Almost all developed countries have a regulatory framework for orphan drugs, i.e., legislative policy or regulations that set the criteria and requirements for orphan drug designation, market authorization, post-marketing monitoring and financial incentives, etc. [3]. Orphan drug policies in these countries have dramatically increased the approval of orphan drugs. The United States (US) was the first nation to introduce orphan drug legislation with the Orphan Drug Act (ODA) of 1983 [4].

In the US, the ODA has been successful in incentivizing manufacturers to research and commercialize orphan drugs for the treatment of a variety of previously underserved conditions including acute myeloid leukemia, chronic lymphocytic leukemia, multiple myeloma, myelodysplastic syndromes, Gaucher disease and Hunter syndrome. Prior to passing of the ODA in 1983, fewer than 10 drugs for orphan conditions were available to patients in the US. In 2013, the Federal Drug and Administration (FDA) website listed more than 400 orphan drugs that have received marketing approval (including both new molecular entities and supplemental applications/indications) [5]. The ODA has served as a model for legislation, and following 1983, a number of other countries subsequently introduced orphan drug policies, including Japan (1993), Australia (1998) and the EU (2000) [6, 7].

Canada is one of the few developed nations without a regulatory framework for orphan drugs and there is currently no official “orphan disease” status in Canada [3]. As a result, patient access has been negatively impacted. Approximately half of approved orphan drugs in the US are available in Canada, with additional variation in access by province [2, 3]. However, Canada has recognized the importance of orphan drug legislation and in October 2012, Health Canada announced the development of a new framework for the designation, authorization and monitoring of orphan drugs in order to benefit Canadians with rare diseases and spur research and innovation in Canada [8].

While orphan drugs have the potential to offer great clinical value and substantial benefits to the patient and to society, payer sensitivity to orphan drugs is increasing. Payers perceive high therapy costs and perceptions of disproportionate use of resources for small patient populations coupled with healthcare budget constraints [9]. However, little evidence or data has been publically generated with regard to actual orphan drug expenditure in Canada. Real-world data is necessary to inform payer and policy discussions on access and reimbursement, as well as to aid in understanding the value of investment in orphan drugs.

This study had several objectives. The first objective was to estimate the financial impact of orphan drugs on the total pharmaceutical expenditure from 2007 to 2013 in Canada. The second objective was to extrapolate orphan drug spend into the future (beyond 2013 and up to 2018).

## Methods

### Study overview

Canada does not currently have an official process for orphan drug designation and thus does not have an official list of drugs with orphan drug designation. Therefore, this analysis used the orphan drugs that have received marketing approval by the FDA in the US as a proxy list of all orphan drugs, and included only those drugs with the orphan indication(s) also approved in Canada. The Health Canada website was used to determine drug approvals and approved indications in Canada. The IMS Health MIDAS database was used to measure orphan drug and total drug expenditure in Canada between 2007 and 2013.

### Data sources

#### Food and Drug Administration (FDA) website

A list of all orphan drugs that have been approved in the US since the passage of the Orphan Drug Act in 1983 up to the end of 2013 was identified from the US FDA Orphan Drug Designations and Approvals Website (*N* = 365 unique products) [5].

Only branded products approved for an orphan indication were included in the analysis (*N* = 356 unique brand name products out of the total 365 brand and generic products). This analysis focused on orphan drug expenditure between 2007 and 2013; however, all drugs approved up to the end of 2013 were considered in order to evaluate all orphan drugs that had received approval prior to 2007, and which continue to factor in orphan drug expenditure during the study analysis period.

#### Health Canada website

The Health Canada website was used to determine and include only those US orphan drug products which received approval in Canada [10]. Because approved indications in the US may differ from those approved in Canada, we additionally evaluated approved indications in Canada, and only considered those products for which the orphan indication or at least one orphan indication (in the case of multiple orphan indications) was also approved in Canada.

#### IMS Health MIDAS database

The IMS Health MIDAS database was utilized to identify sales for the identified branded, orphan drugs [11]. MIDAS integrates and extends IMS National Audits, the IMS ‘building blocks’ which accurately detail estimated product volumes, trends and market share by product and therapy class, through retail and non-retail channels. Sales data are collected at the unit level of the pack and identifiable information for each pack includes product name, manufacturer name, dose form (e.g., tablets), dose strength (e.g., 50 mg), and number of doses (e.g., 20 tablets). In Canada, MIDAS tracks the direct sales (i.e., sales invoices) of pharmaceuticals from the manufacturer to pharmacies or hospitals. MIDAS also tracks indirect sales (sales going through a middleman i.e., the wholesaler) to pharmacies and hospitals. MIDAS tracks inflow or what these different channels are purchasing (i.e., the sales made into those outlets). MIDAS represents the full Canadian market through representative panel projections for both retail and hospital channels. The data are routinely used by governments and academics in Canada to best understand market volumes and growth.

### Partial orphans

Since a drug may be approved for multiple indications, further evaluation was undertaken for “partial orphans”, or those products with both orphan and non-orphan indications. Of the 356 branded orphan drugs designated by the FDA, 65 (18 %) were identified as partial orphans in the US.

MIDAS sales data does not comprehensively segment by indication. In a parallel analysis of orphan drug expenditure in the US, in-depth analysis was conducted for the partial orphan drugs in order to determine an indication factor to apply to the sales for each partial orphan product to isolate and include only orphan indication(s) sales. Since sources for indication factors were limited in Canada (limited data sources or financial reporting; smaller population size, particularly for orphan diseases, etc.), indication factors identified for the US analysis were used in Canada as a proxy. Approval dates for orphan and non-orphan indications in Canada were also considered as applicable.

In the US analysis to determine an indication factor for each partial orphan, to estimate the proportion of total drug sales spent on the orphan indication(s), several US sources were consulted including the Cowen and Company Equity Research Health Care Therapeutic Categories Report published March 2014 and manufacturers’ audited financial reports [12]. The Cowen Report included relevant information for some orphan drugs such as estimates of therapy area sales by product (e.g., US Rheumatoid Arthritis Market Model) or product sales by indication (e.g., Abraxane Pancreatic Cancer Revenue Model). Additionally, an analysis of US physician office claims data was conducted in order to assess diagnoses associated with prescriptions among a large sample of US physicians, to quantify the number of orphan diagnoses associated with prescriptions of a partial orphan drug. Similarly, another source available was the diagnosis value in MIDAS. The diagnosis value is derived from a survey that is administered to office-based physicians and is a measure of physician-reported indications for prescribed drugs. Specifically, it calculates the proportion of prescriptions for a product form strength that is prescribed for a diagnosis. It is applicable to drugs prescribed from office-based physicians but limited for any hospital-based drugs. Lastly, reported epidemiology (incidence or prevalence rates) in the US was used to determine the different disease population proportions. All available sources were evaluated with the indication factor from the most robust source (taking into account sample size [number of claims], setting of care, etc.) being applied to the total product sales in order to measure sales associated with orphan indications. Approval dates were also considered as applicable.

### Measurements

Total orphan drug expenditure was calculated annually from 2007–2013 and further reported as a proportion of total annual pharmaceutical drug expenditure, also derived from MIDAS. Expenditure was recorded in Canadian dollars (CAD) at the time of the sale. All expenditure was adjusted to 2014 CAD using the Canadian Consumer Price Index [13]. Trends in total orphan drug expenditure were assessed over time. In an exploratory analysis, major therapeutic categories of orphan indications were examined and expenditure for orphan drugs with oncology indications was evaluated, and reported, as a subset of all orphan drugs.

### Future trend analysis

A future trend analysis was evaluated to estimate orphan drug sales from 2014–2018. Orphan drug sales from 2007–2013 were used to project 2014–2018 sales. Because we observed a gradual increase in orphan drug spending over time from 2007–13 (i.e., a generally consistent linear trend), suggesting that the orphan drug segment has historically been relatively stable, we used a linear trend model. While various factors impact future orphan drug spending, we believe this was an appropriate option given the lack of data needed for a more complex analysis (e.g., no official orphan drug approval rate in Canada). Future estimated orphan drug expenditure was evaluated as a proportion of projected future total drug expenditure in Canada based on the IMS Health Market Prognosis forecasts [14]. IMS Market Prognosis is a strategic market forecasting publication that combines historical data and macroeconomic indicators with the identification and evaluation of key future issues to feed into an econometric model. IMS Market Prognosis provides a 5-year forecast of total market sales based on rigorous evaluation of the key events affecting the marketplace (e.g., legislative changes, improved market access for orphan drugs, major new product launches, cost-containment measures, generic competition, etc.). Market Prognosis provides a global pharmaceutical outlook with 49 in-depth country forecasts. Because the Market Prognosis forecast for total pharmaceutical spend includes three categories: Brand name prescriptions, Generic prescriptions, and Other (over-the-counter [OTC], non-categorized Rx and other Rx; which accounted for 14 % of the total pharmaceutical spend in 2013), the annual growth rate for brand and generic prescriptions from the forecast was applied to the 2013 MIDAS data for total pharmaceutical expenditure, to calculate adjusted total pharmaceutical expenditure for prescription pharmaceuticals only for each year from 2014–2018.

## Results

Of the 356 branded products identified from the FDA website, MIDAS captured sales for 147 unique orphan drugs in Canada between 2007 and 2013, of which, 39 (26.5 %) were partial orphan drugs. Reasons for sales data not being available include the drug not having been approved in Canada, the orphan indication in the US not being approved in Canada, funding for the drug being unavailable in Canada, the product no longer being marketed in Canada or other reasons, such as MIDAS coverage limitations (i.e., infrequent cases where sales are not audited). In our review of the Health Canada website to identify “matches” for US orphan drugs, we found a match to a product in Canada (i.e., a Notice of Compliance [NOC]) for 207 orphan drugs. Of these 207 orphan drugs, 11 orphan drugs did not have the orphan indication approved in Canada, and were not included in the final sample for analysis. We do not know the reasons for the MIDAS data not including 49 other orphan products with a NOC in Canada. It is possible that these drugs have been discontinued or have not actually been marketed in Canada.

**Fig. 1:**
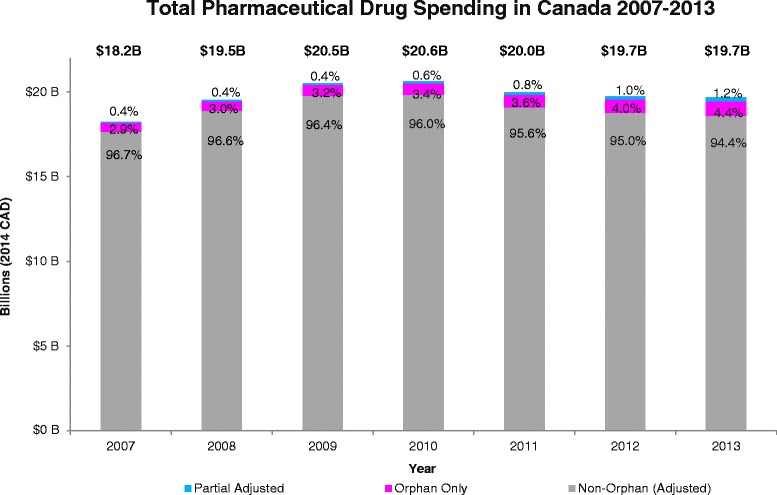
2007–2013 Orphan Drug Expenditure out of Total Canadian Drug Expenditure

Following indication factoring and adjustment for partial orphan drugs, total orphan drug expenditure totaled $610.2 million (M) in 2007 and $1,100.0 M in 2013, representing 3.3– 5.6 % of total Canadian pharmaceutical drug expenditure in 2007 and 2013, respectively (Table 1; Fig. 1). Orphan only drugs represented 2.9– 4.4 % of total Canadian drug sales from 2007–2013. Partial orphan drugs represented 0.4–1.2 % of total Canadian drug sales from 2007–2013.

**Table 1:** 2007–2013 Canadian Orphan Drug Landscape and Expenditure (2014 CAD, Millions)

Measure	2007	2008	2009	2010	2011	2012	2013
# “Orphan only” drugs captured *N* = 108	70	74	73	79	81	91	99
# “Partial Orphan” drugs captured *N* = 39	25	26	27	28	30	31	34
# Total Orphan drugs captured *N* = 147	95	100	100	107	111	122	133
“Orphan Only” Drug Expenditure ($M)	537.6	586.7	649.2	692.6	729.3	786.8	868.0
“Partial Orphan” Drug Expenditure ($M)	72.6	82.5	94.5	125.5	151.2	202.8	232.0
Total Orphan Drug Expenditure ($M)	610.2	669.2	743.7	818.1	880.5	989.6	1,100.0
% “Orphan Only”/Total Orphan Expenditure	88.1 %	87.7 %	87.3 %	84.7 %	82.8 %	79.5 %	78.9 %
% “Partial Orphan”/Total Orphan Expenditure	11.9 %	12.3 %	12.7 %	15.3 %	17.2 %	20.5 %	21.1 %
Total Pharmaceutical Expenditure ($M)	18,233.6	19,598.1	20,514.4	20,628.6	19,976.3	19,746.0	19,665.7
% Total Orphan/Total Pharmaceutical Expenditure	3.3 %	3.4 %	3.6 %	4.0 %	4.4 %	5.0 %	5.6 %

While orphan drug expenditure increased over time, it is important to note that the number of orphan drugs approved and reimbursed in Canada has also increased over time, with MIDAS capturing sales for a total of 95 unique orphan drugs in 2007, which increased to 133 orphan drugs in 2013.

Major therapeutic categories of indications were evaluated and oncology was the most common therapeutic class, with 57 (38.8 %) out of 147 orphan drugs identified with an orphan oncology indication. Expenditure for these oncology-related orphan drugs totaled $227.8 M in 2007 and $474.7 M in 2013, representing 37.3 and 43.2 % of total orphan drug expenditure, respectively.

**Fig. 2:**
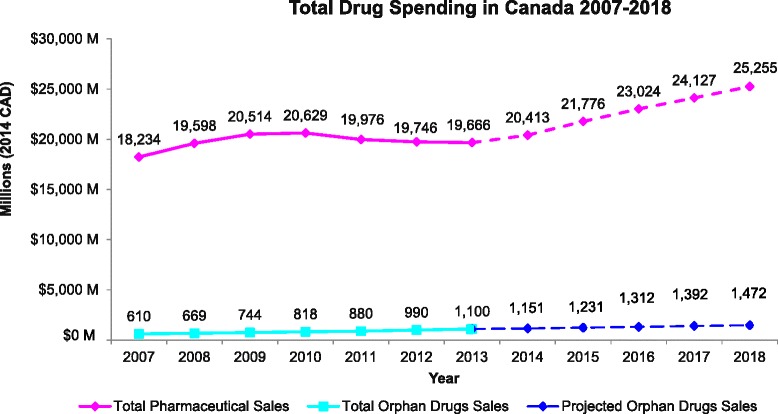
Current (2007–2013) and Future (2014–2018) Orphan Drug Spending

**Fig. 3:**
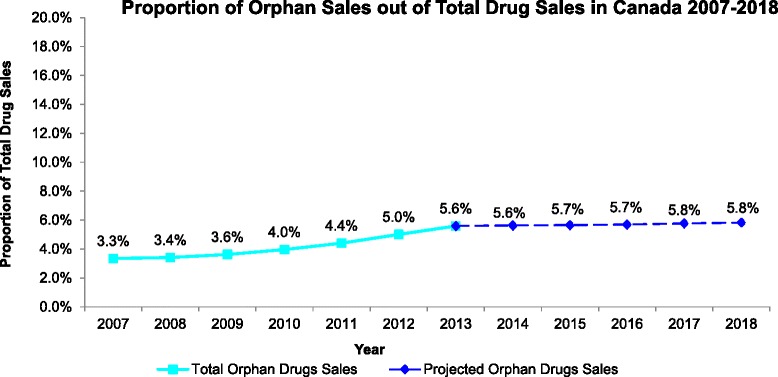
Current (2007–2013) and Future (2014–2018) Total Drug Spending

In the future trend analysis, a linear trend line was created using 2007–2013 sales (in millions), with equation y = 80.253x + 509.16, and with R^2^ = 0.9871, resulting in sales for 2014 to 2018 of $1,151.2 M to $1,472.2 M, respectively, representing a 27.9 % increase between 2014 and 2018 (Fig. 2). IMS Health Market Prognosis forecasts were adjusted to reflect prescription pharmaceuticals only, and 2014–2018 Canadian total drug sales were estimated at $20,413 M to $25,255 M, representing a 23.7 % increase from 2014 to 2018. Thus, the projected orphan drug sales represent 5.6 to 5.8 % of total Canadian drug expenditure from 2014 to 2018, respectively (Fig. 3).

## Discussion

The ODA led to an increase in the number of approved orphan drugs in the US, which also led to an associated increase in the number of drugs available to benefit many people with previously underserved rare conditions in Canada. While associated orphan drug expenditure has increased, the population using these orphan drugs is relatively small. Total orphan drug expenditure, when considered out of total drug expenditure, is comparatively low, representing less than 6 % of total pharmaceutical expenditure. While both total orphan drug and total drug expenditure are forecasted to increase, future trend analysis suggests that orphan drug expenditure in 2014–18 will remain stable below 6 % as a proportion of total drug expenditure. This may be related to drug spending accounting for a declining share of total health dollars, as reported by the Canadian Institute for Health Information in 2013 [15]. To aid in the interpretation of our study findings, in a broader context, drug expenditure represents a minority share when considered out of total health care expenditure. For example, in 2013, total spending on drugs (including non-prescription drugs [e.g., OTC]) was estimated to represent only 16 % of the total $211 billion spent on health care in Canada, and drug spending growth (2.4 %) was slower compared to both hospital spending (2.6 %) and physician spending (3.6 % growth) [15].

Orphan drug policies in other countries, such as the ODA in the US, were created to address the unmet need for developing therapies to treat rare diseases. Canada is now following these trends, and is currently developing orphan drug policies. Orphan drug policies have led to an increase in the number of drugs to treat patients with rare or orphan diseases in those countries, as well as in Canada. In our analysis, MIDAS captured 95 orphan drugs in 2007 which increased to 133 orphan drugs in 2013. However, we found a significant disparity in the availability of orphan drugs in Canada when we compared the number of unique orphan drugs identified in Canada to the number identified in the US during the same time period. In our parallel US analysis, we captured sales in MIDAS for a total of 316 unique orphan drugs between 2007 and 2013 (out of the total 356 approved unique branded orphan drugs). Only a total of 147 unique orphan drugs were captured in Canada in MIDAS during the same time period demonstrating the disparity in access, consistent with other reports that more than half of the orphan drugs approved in the US are not available in Canada [2, 3].

In our analysis, a quarter of available orphan drugs (39/147) were also indicated to treat non-orphan conditions. This is often not mentioned in critiques of the costs of orphan drugs, and our indication factoring analysis suggests that the majority of unadjusted “partial orphan” drug sales are for non-orphan indications. Adjusted “partial orphan” drug expenditure represented only 5.6– 9.1 % of unadjusted “partial orphan” drug expenditure from 2007–2013.

While our analysis focused on orphan drug expenditure, it is important to consider that these orphan drugs benefit many people with previously underserved rare conditions. Furthermore, these drugs offer significant value to both patients and society such as improvements in health, survival and quality of life, reductions in costly hospitalizations, reduced disabilities, and increased ability to go back to work, resulting in increased productivity. The findings from this analysis suggest that investment in drugs for orphan diseases appears to be justified in Canada. According to the Canadian Organization for Rare Disorders, an estimated 9 % of Canadians suffer from a rare disease. This has been termed as a “paradox of rarity”, in which an individual disease may be rare, but a significant proportion of a country’s population may be affected by a rare disease [16]. Interestingly, expenditure on orphan drugs represented less than 6 % of total pharmaceutical expenditure in Canada from 2007–2013. Although spending on an individual patient level may seem disproportionate compared to drug spending on the individual basis for non-orphan drugs, from the perspectives of society and equity, overall spending on orphan drugs is low, considering the number of patients affected with an orphan disease in Canada, and access remains an issue.

We did not identify any other published studies that evaluated the costs of orphan drugs in Canada. However, our parallel analysis in the US found orphan drug expenditure was generally higher in the US than in Canada (4.8– 8.9 % of total US drug expenditure between 2007 and 2013), while both future trend analyses similarly suggested slowing down in the growth of orphan drug expenditure. Furthermore, a few recent studies have examined the budget impact of orphan drugs in Europe [17–20]. Although methods and data sources varied (based on sales data, disease-based epidemiological models, etc.) limiting comparisons, findings suggest that orphan drug expenditure as a percentage of total pharmaceutical sales in Europe and Canada is comparable. Estimates ranged from 2.5 % in Sweden and 3.1 % in France in 2012 [17], 1.6– 4.2 % from 2007–2012 in the Netherlands [18], 1.9– 4 % from 2008–2013 in Belgium [19] and 3.3 % in 2010 in Europe (Eurozone + UK) [20], with growth expected to slow or peak and level off. Comparisons are further limited due to varying country-specific access and reimbursement guidelines and policies.

## Limitations

There are a number of limitations with this analysis. MIDAS database coverage is limited in some cases, for a few individual specialty products which may have unique distribution arrangements which prevent IMS from auditing their sales. However, this is infrequent and this does not impact the audit at the national level for the majority of products. This limitation could result in an underestimation of orphan drug sales. Furthermore, IMS applies custom methodologies as needed to ensure projections are as valid as possible. Additionally, the future trend analysis does not account for changing trends following final approval and implementation of the orphan drug framework in Canada, which could result in an underestimation of forecasted orphan drug spending. While various factors impact future orphan drug spending, we chose a linear trend model for the future analysis, given the lack of data needed for a more complex analysis. The future trend analysis relied on historical expenditure and historical trends and does not account for any potential heightened focus of the pharmaceutical industry on R&D for orphan drugs. However, our R^2^ was quite high, and suggests our model may be a good fit and may explain much of the variance. Our analysis also does not focus on any specific high-cost “blockbuster” orphan drugs and instead considered overall orphan drug costs. As Canada is currently developing an orphan drug framework, orphan drugs approved by the FDA in the US were used as a proxy for the orphan drug landscape in Canada. Differences in indication approvals may vary between the US and Canada. While indication differences were reviewed to ensure US orphan indication(s) were also approved in Canada, it is a limitation that any additional indications approved in Canada, but not approved in the US were unaccounted for. Additionally, there is the potential for off-label use of orphan drugs as the sales data do not have indications associated with sales. Not thoroughly excluding non-orphan drug sales may result in an overestimation of total orphan drug sales. However, prior authorization or special authorization is often used by payers as a condition for reimbursement which minimizes the potential for off-label use, especially for high cost drugs. Finally, our analysis focused on brand orphan drugs only, including those approved pre-2007. It is possible that any generics of orphan drugs that have become available have eroded brand market share and also contribute to the orphan drug budget, although at a steep discount; on average, costs of generics are from 80– 85 % lower than brand products [21]. Thus, this may result in a slight underestimation.

## Conclusions

The analysis shows that total spending on orphan drugs represents a small share of overall pharmaceutical spending and the overall budget impact of orphan drugs has remained fairly stable relative to total pharmaceutical expenditure. Future orphan drug spending is estimated to remain low in relation to overall pharmaceutical spending. Concerns that growth in orphan drug expenditure may lead to unsustainable drug expenditure do not appear to be justified.
